# Specialized Feeding Behavior of an Endangered Primate Enhances Forest Health in China

**DOI:** 10.1002/ece3.72810

**Published:** 2026-01-24

**Authors:** Na Li, Hao‐Han Wang, Yan‐Peng Li, Cyril C. Grueter, Hui‐Ming Xu, Zhi‐Pang Huang, Wen Xiao

**Affiliations:** ^1^ Institute of Eastern‐Himalaya Biodiversity Research, Dali University Dali Yunnan China; ^2^ Yunling Black‐and‐White Snub‐Nosed Monkey Observation and Research Station of Yunnan Province Dali Yunnan China; ^3^ Collaborative Innovation Center for Biodiversity and Conservation in the Three Parallel Rivers Region of China Dali Yunnan China; ^4^ The Provincial Innovation Team of Biodiversity Conservation and Utility of the Three Parallel Rivers Region From Dali University Dali Yunnan China; ^5^ Center for Interdisciplinary Sciences, Dali University Dali Yunnan China; ^6^ Research Center of Natural History and Culture, Qujing Normal University Qujing Yunnan China; ^7^ School of Human Sciences, the University of Western Australia Perth Western Australia Australia; ^8^ International Centre of Biodiversity and Primates Conservation Dali Yunnan China; ^9^ Bureau of Tianchi National Nature Reserve Dali Yunnan China

**Keywords:** biological interaction, ecosystem management, forest dependent, lichen, parasite, rare primate, specialized diet

## Abstract

A study on the long‐term ecological impacts of specialized behavior can provide valuable insights for biodiversity conservation and ecosystem management, particularly in the context of climate change. The endangered primate *Rhinopithecus bieti* in temperate forest ecosystems relies primarily on the lichen *Usnea longissima* as its fallback food source. To investigate whether this specialized diet sustains forest health through trophic interactions, our study employed a three‐tiered approach: (1) We first examined the impact of *U. longissima* on trees by comparing the health of branches covered with and without lichen. Findings reveal *U. longissima* exhibits harm to host trees, as lichen‐covered branches displayed significantly higher rates of dieback. (2) Using habitats with varying extinction timelines of *R. bieti* , we quantified how the presence of *R. bieti* contributes to reducing *U. longissima* biomass. Results showed lichen biomass tripled in habitats where the species vanished 40 years ago compared to occupied habitats. (3) We finally used controlled artificial experiments that demonstrated that *R. bieti's* feeding activities may enhance *U. longissima* dispersal and growth. Our findings suggest that *R. bieti* may function as a natural regulator of lichen biomass, potentially helping to prevent overgrowth that could destabilize forest health. Notably, the monkeys' foraging behavior may not only control lichen proliferation but also help to promote its regeneration. This study underscores that restoring *R. bieti* populations would synergistically benefit both *U. longissima* viability and forest resilience, advocating for integrated conservation strategies that preserve specialized ecological interactions. Due to the fact that specialized diet species face severe survival challenges in the context of climate and environmental changes, future efforts should be focused on their ecological adaptation mechanisms and improving sustainable management strategies.

## Graphical abstract

The endangered black‐and‐white snub‐nosed monkey ( *Rhinopithecus bieti* ) exhibits a specialized diet on the lichen *Usnea longissima* . We proved that the *U. longissima* is harmful to trees and the monkey mitigates overgrowth of the lichen, as well as promotes its regeneration to maintain population, thereby promoting ecosystem health and resilience. This finding underscores that specialized behaviors may fulfill critical ecological roles, and should be incorporated in ecosystem‐based conservation strategy.

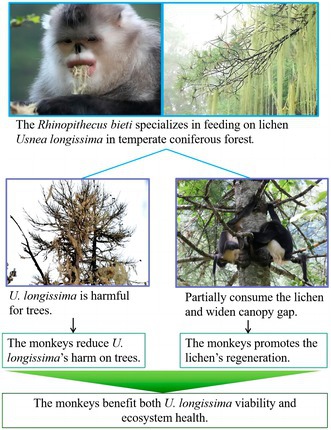

## 1 Introduction

The specialization of biological behaviors and traits is the core mechanism driving ecological niche differentiation, distribution patterns, and evolutionary processes of species (Amadon 1943; Vamosi et al. 2014). Among them, dietary specialization, as the most typical research direction, provides key adaptive strategies for species survival and coexistence by allowing them to utilize “fallback food” in resource‐poor environments (Brandl et al. 2015). For instance, the co‐evolution between hummingbirds and flowers (Barreto et al. 2024), the high dependence of koalas on eucalyptus leaves (Johnson et al. 2018), the specific feeding on bamboo by giant pandas (Wei et al. 2015), the specialized predation on ants by anteaters (Gaudin et al. 2018), and the seasonal dependence of reindeer on lichens in Arctic tundra (Dominy et al. 2023), all illustrate the crucial role of dietary specialization in constructing the ecological niche of species. Moreover, studies on specialized diets among different species or individuals in bird and fish communities have revealed how this mechanism optimizes resource utilization and maintains community stability (Brandl et al. 2015; Dehling et al. 2016). However, though previous studies have extensively documented the critical role of specialized foraging behaviors in securing species survival, and a few studies have explored localized impacts of specialized feeding on food resources (Wang et al. 2020), a striking gap persists in understanding how these behaviors propagate ecological consequences beyond individual or population scales to influence entire ecosystem processes (Clavel et al. 2011). Systematic investigations into ecosystem‐level cascades—such as trophic interactions and long‐term stability—remain alarmingly scarce. This knowledge deficit risks grossly undervaluing the role of foraging specialization in sustaining ecosystem multifunctionality, thereby undermining efforts to design holistic conservation strategies.

Moreover, forest ecosystems, as keystone providers of global climate regulation and carbon sequestration (Pan et al. 2024), face mounting threats from climate change. By dissecting the long‐term ecological ripple effects of specialized foraging behaviors within these systems, our work addresses this critical gap. It offers mechanistic insights into how behavioral adaptations stabilize ecosystem services, ultimately providing actionable frameworks for climate‐resilient conservation policies. In an era of escalating biodiversity loss and habitat fragmentation, this integration of behavioral ecology with ecosystem science is imperative to safeguard the interdependent relationships that underpin both ecological stability and human well‐being.

**FIGURE 1:**
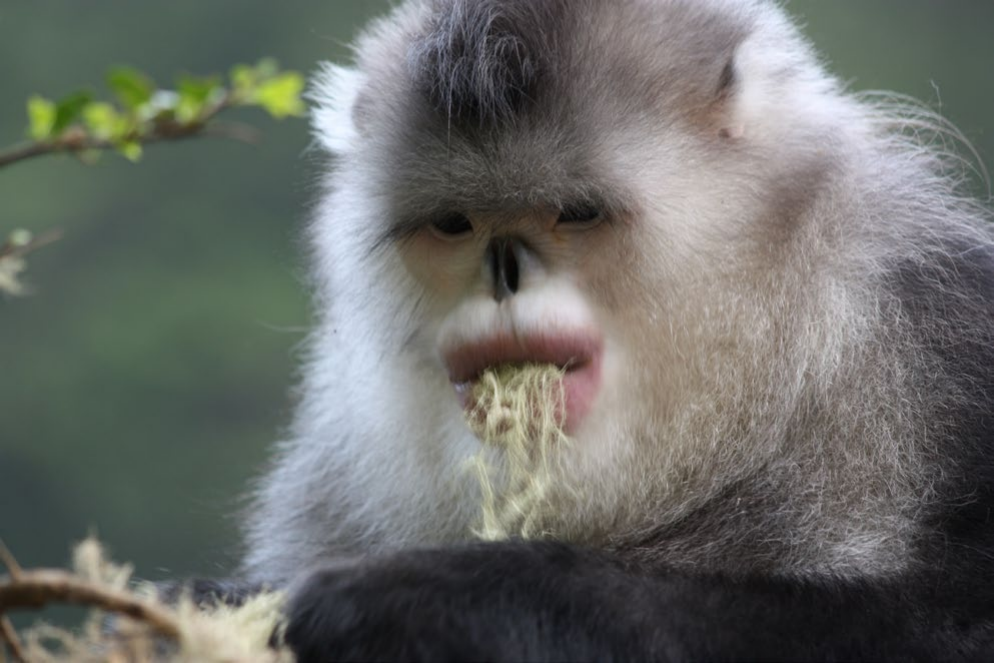
The black‐and‐white snub‐nosed monkey ( *Rhinopithecus bieti* ) feed on lichen *Usnea longissima* . The lichen is the main fallback food of the monkey's diet, and this specialized feeding relationship occurs in the temperate coniferous forest ecosystem in high and cold elevations.

Therefore, this study focuses on the specialized diet of the black‐and‐white snub‐nosed monkey *Rhinopithecus bieti* on lichen *Usnea longissima* in forests (see Figure 1), and explores the ecological impacts of this specialized behavior. The *U. longissima* is a typical lichen as a large flagship species for biodiversity conservation in forests (Esseen et al. 1992, 2023), and is regarded as a symbol of virgin forests, and a widely used environmental indicator (Esseen et al. 2022). This lichen is hair‐like may reach a length of several meters, constituting the longest lichen in the world. *U. longissima* is distributed in most part of Europe, the Ural area, the Himalaya, China, Taiwan, the Philippines, Japan, and Papua New Guinea (Wei 1991; Brodo et al. 2001; Clerc 2011; Esseen et al. 2023). The endangered snub‐nosed monkey (Yongcheng et al. 2020) is distributed in a narrow range in the Mountains of Southwest China biodiversity hotspot (Xiao et al. 2003) with a total number of 3500 individuals surveyed by our research group in 2021 (unpublished data). The monkey has been advocated as a surrogate species for biodiversity conservation. The lichen *U. longissima* and *Bryoria* spp. compose ~80% of the monkey's diet (Huang et al. 2017), serving as their primary fallback food throughout the year. This reliance occurs because their preferred high‐quality seasonal food items—including young leaves, flowers, and fruits/seeds—are both quantitatively limited and temporally restricted to specific seasons (Huang et al. 2017). Occupy relatively high and cold elevations (Grueter et al. 2012), this specialized feeding relationship occurs in fragile yet critical ecosystems: the temperate coniferous forest ecosystem (Xiao et al. 2003). Research into the long‐term ecological impacts of this specialized dietary relationship is scientifically significant for the sustainable management of these ecosystems.

We assume that the foraging behavior of *R. bieti* on *U. longissima* could maintain the health of the forest by controlling the biomass of *U. longissima* . We hypothesize that: (1) the monkey's main diet lichen *U. longissima* was harmful to trees, that is it may be a parasite rather than the previously assumed epiphyte; (2) the monkey *R. bieti* reduced the harm of its main diet *U. longissima* on the forest by controlling the lichen's biomass; (3) the monkey promotes the regeneration of *U. longissima* population, thus in turn maintaining its own population. Previous studies mentioned that the *U. longissima* are often found on dead branches of live host trees or in the tops of dead trees (Wetmore 2002), and live trees hosting *U. longissima* were found dead after decades (Esseen et al. 2023). We also found the host trees dead based on decades of wild observation. However, the causal relationship between *U. longissima* presence and tree mortality remains to be established, as tree death could result from multiple factors. So we first test if *U. longissima* is harmful to trees by measuring the influence of this lichen on colonized trees in the field based on control experiments. We then test the hypothesis that *R. bieti* can control the lichen's biomass by comparing the biomass in a series of historical habitats with gradually local extinction times. We finally test if the foraging behavior of *R. bieti* could promote the regeneration of *U. longissima* through field measurements and a series of artificial simulating experiments.

If the hypothesis is proved, the monkey *R. bieti* will be redefined as a key ecological regulator in temperate coniferous forests. Its foraging behavior will maintain the forest's carbon sink capacity and species coexistence by curbing the excessive growth of lichens. This discovery will provide a scientific basis for the sustainable management of alpine ecosystems and endangered species conservation: while protecting the monkey, its behavior will indirectly maintain the health of the forest ecosystem. This study would give insight into specialized behavior and consequently inspire a new perspective of sustainable ecosystem management.

## 2 Materials and Methods

### 2.1 Study Area

**FIGURE 2:**
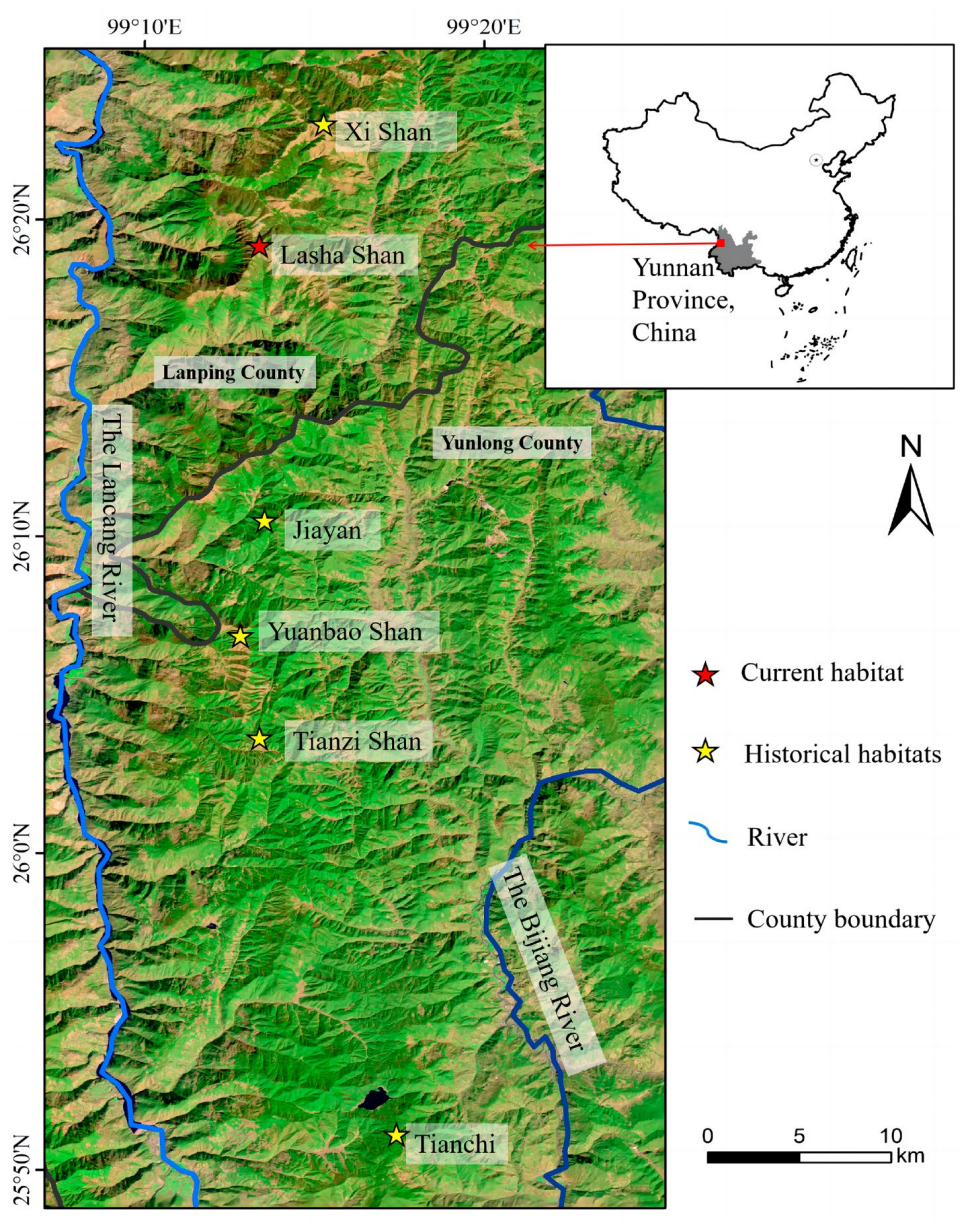
Study area showing the research sites of current habitat and historical habitats of *Usnea longissima* 's forager, the black‐and‐white snub‐nosed monkey ( *Rhinopithecus bieti* ). Lasha Shan constitutes the current habitat (H‐0). The population at Jiayan (H‐10) vanished approximately 10 years before 2019, and those at Yuanbao Shan (H‐20), Tianzi Shan (H‐30), Tianchi (H‐40), and Xi Shan (H‐40) became extirpated approximately 20, 30, 40, and 40 years ago, respectively.

The study area (25° 50′N–26°25′N; 99°15′ E) is located northwest of Yunnan Province, China (Figure 2). It lies at the intersection of three global biodiversity hotspots: the Himalayas, the Indo‐Myanmar region, and the mountains of Southwest China (Zachos and Habel 2011), as one of the richest biodiversity regions in the world. The study area is 50 km long from north to south and 3 km wide from east to west, on the east of the Lancang River and on the Yunling Mountains. The climate and vegetation characteristics within the study area are similar, which encompass temperate coniferous forests where the *U. longissima* is the dominant canopy lichen, and are characterized by a vegetation layer including *Tsuga dumosa, Abies georgei, Rhododendron faberi, Rhododendron mucronatum*, and *Gamblea ciliata* var. *evodiifolia*. Mean annual rainfall in the area is 1000–1800 mm, which falls in a strongly seasonal fashion, with a rainy season (June–October) and a dry season (November–May).

The study area contains the forager monkey's current habitat, that is Lasha Shan, but also historical habitats, that is Jiayan, Yuanbao Shan, Tianzi Shan, and Tianchi and Xi Shan. The population size in Lasha Shan was around 100 individuals and home range was about 40 km^2^ in 2024. Based on our interview‐based surveys conducted in 2019, the monkey groups in these historical habitats vanished at different times: 2008 at Jiayan 1997 at Yuanbao Shan, 1989 at Tianzi Shan, and 1980 at Tianchi and Xi Shan. So we denote these habitats as H‐0, H‐10, H‐20, H‐30, and H‐40 in sequence of the monkey's local extinction years. These study sites were selected due to three key characteristics: (a) clearly definable activity ranges of monkey groups, (b) homogeneous geological and climatic conditions (Table 1), and (c) minimal human disturbance and no vegetation structural changes resulting from natural disasters. Under these controlled environmental parameters, the method of spatial–temporal substitution becomes applicable. This approach allows us to infer temporal ecological dynamics by analyzing spatial gradients, thereby validating our hypothesis through a proxy‐based framework.

**TABLE 1:** The geography, climate, and vegetation composition in current and historical habitats.

Habitats	Elevation (m)	Annual precipitation (mm)	Mean temperature (°C)	Trees composition	Shrubs composition
				Species name (abundance)	Species name (abundance)
H‐0	1520–3845	1300	13	*Abies georgei* (61), *Acanthopanax evodiaefolius* (45), *Tsuga dumosa* (37), *Rhododendron mucronatum* (23), *Symplocos heishanensis* (21)	*Fargesia dura* (53), *Fargesia spathacea* (44), *Rhododendron faberi* (11), *Nothopanax delavayi* (9), *Berberis mitifolia* (8)
H‐10	1420–3412	1300	13	*Tsuga dumosa* (91), *Larix gmelinii* (61), *Rhododendron faberi* (34), *Rhododendron oreodoxa* (34), *Lithocarpus variolosus* (27)	*Fargesia papyrifera* (36), *Fargesia spathacea* (32), *Rhododendron faberi* (26), *Lonicera buchananii* (9), *Berberis mitifolia* (9)
H‐20	1470–3475	1300	13	*Tsuga dumosa* (78), *Abies georgei* (51), *Lithocarpus variolosus* (40), *Acanthopanax evodiaefolius* (35), *Rhododendron faberi* (24)	*Rhododendron faberi* (36), *Fargesia spathacea* (33), *Fargesia papyrifera* (33), *Berberis mitifolia* (18), *Cotoneaster acuminatus* (14)
H‐30	1489–3423	1200	12	*Tsuga dumosa* (86), *Abies georgei* (68), *Rhododendron faberi* (41), *Acanthopanax evodiaefolius* (29), *Lithocarpus variolosus* (23)	*Rhododendron faberi* (45), *Fargesia papyrifera* (36), *Fargesia spathacea* (19), *Berberis mitifolia* (18), *Cotoneaster acuminatus* (16)
H‐40	1419–3518	1500	12	*Abies georgei* (60), *Tsuga dumosa* (43), *Rhododendron faberi* (38), *Acanthopanax evodiaefolius* (28), *Rhododendron mucronatum* (21)	*Fargesia spathacea* (37), *Rhododendron faberi* (27), *Fargesia dura* (22), *Berberis mitifolia* (18), *Cotoneaster horizontalis* (14)

*Note:* Vegetation composition is shown as the five most abundant trees and shrubs. Abundance refers to the number of stems collected in the quadrats from the five habitat sites.

### 2.2 The Effect of *Usnea longissima* on the Health of Trees

**FIGURE 3:**
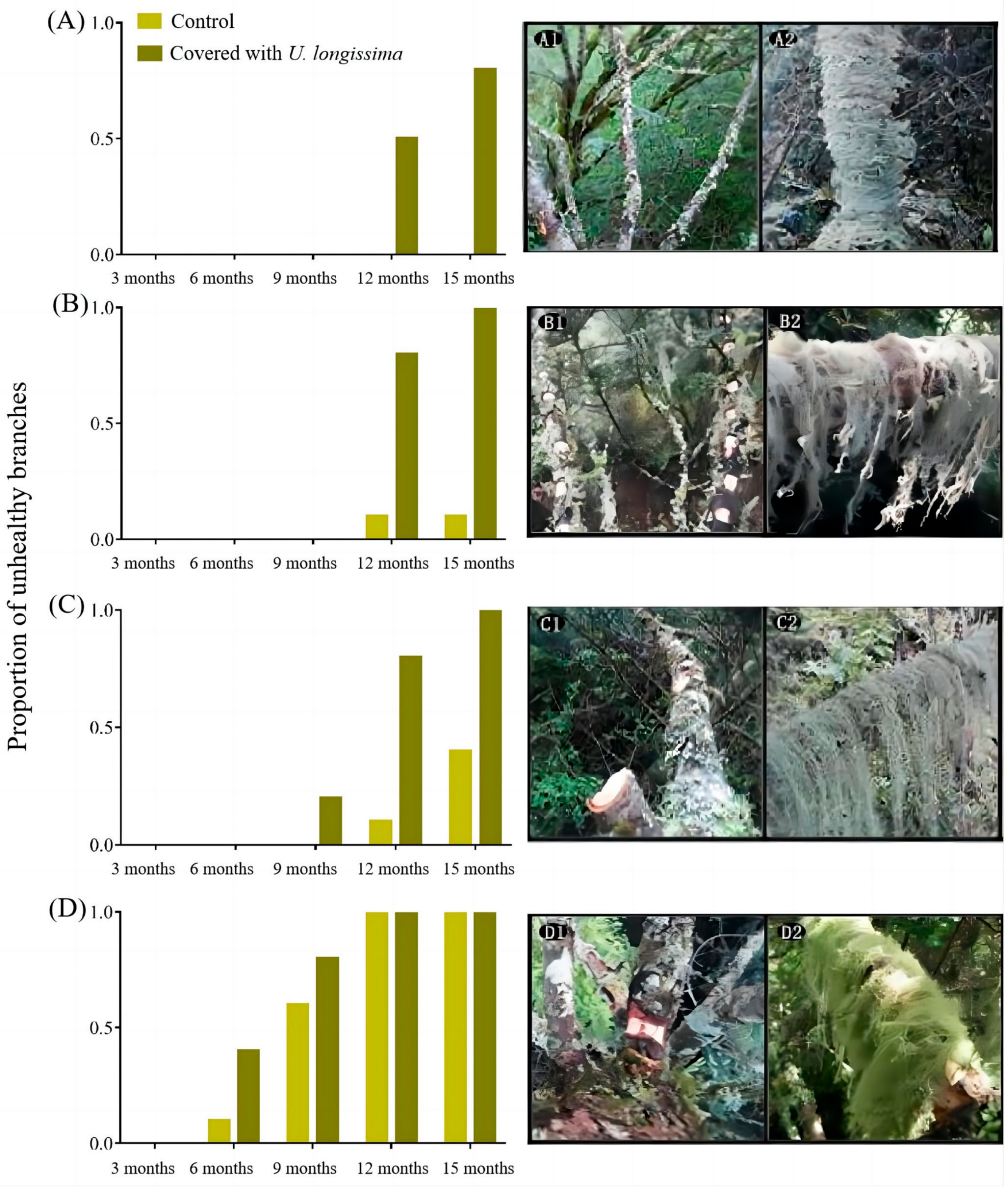
Temporal progression of unhealthy branch proportions in control and *U. longissima* ‐covered groups under varied treatments. The proportion of unhealthy branches increased over time and branches covered with *U. longissima* were more harmed in all groups, and in groups A, B, C, and D, unhealthy branches increased in turn. A1‐D1 are the control group and A2‐D2 are the experimental group. (A) A1‐No treatment on the healthy main branch; A2‐ *U. longissima* on the healthy main branch. (B) B1‐No *U. longissima* on the main branch with two‐thirds of the bark left; B2‐ *U. longissima* on the main branch with two‐thirds of the bark left. (C) C1‐No *U. longissima* on the main branch with all the side branches pruned; C2‐ *U. longissima* on the main branch with all the side branches pruned. (D) D1‐No *U. longissima* on the main branch with the bark ringed at the root; D2‐ *U. longissima* on the main branch with the bark ringed at the root.

First, we conducted a controlled field experiment for 15 months to test the effect of *U. longissima* on the health of the host trees and to make sure whether it is an epiphyte or parasite. We set up four 5 × 5 m quadrats in Jiayan corresponding to four treatments: (a) no treatment; (b) removing one third of the bark by cutting square notches in the main branches; (c) pruning the side branches; (d) girdling the roots of the main branches (Figure 3A–D). In every quadrat, we randomly chose four coniferous trees and divided them into two groups, that is the experimental group and the control group, each of which contained two trees. On each tree, we chose five healthy main branches with a diameter of approximately 7–10 cm. In the experimental group, we hung *U. longissima* with a length of 30 cm on the selected branches and made sure that the branches were 100% covered. We ensured that *U. longissima* did not touch the tree leaves. This was done to prevent disruption of the photosynthesis process. We kept the branches in the control group free from *U. longissima* . All quadrats were monitored every 3 months for a period of 15 months to record the health status of branches and *U. longissima* , so 5 monitored periods were included in total. We categorized branches as unhealthy if more than 10% of the leaves on the branch were yellowing or shedding. Dried and rotten branches were considered dead. When a monitored branch was dead, the monitoring process on the branch would stop.

### 2.3 The Consumer Monkey's Feeding Behavior Record

We then quantified the snub‐nosed monkeys' feeding behavior on the quantity of *U. longissima* using focal observations and field measurement. Feeding on *U. longissima* by the monkey at Lasha Shan was recorded from October 2018 to May 2019. We set up 10 quadrats (20 m × 20 m) in areas of frequent use and with high visibility. Of these, three were on northern slopes (3300, 3400, and 3500 m a.s.l.), four on eastern slopes (3200, 3300, 3400, and 3500 m a.s.l.), and three on western slopes (3200, 3400, and 3500 m a.s.l.). We randomly selected five tall trees (3 conifers and 2 broad‐leaved trees) from each of the ten quadrats. Considering that the monkeys may feed on the lichen of different parts of a tree in different ways, the canopy of each tree was vertically divided into three layers and horizontally into two layers. First, we measured the diameter of the tree canopy by calculating the area of its projection on the ground. Then, we divided it into two equal parts: the inner and outer layers. We climbed up to measure the position of the lowest branches of the tree crowns and the height of the trees, calculated the canopy height, and divided it into three equal parts: upper, middle, and lower layers. The canopy was also divided into four directions: east, west, south and north. Each tree was thus divided into 24 segments. A small quadrat with a size of 5 × 5 cm was randomly placed in each segment. When setting up the small quadrats, we determined our position within the layers by measuring the distance between ourselves and the tree trunk, as well as the position of the lower edge of the tree canopy. There were 1200 small quadrats (24 segments trees×10 quadrats). Different filaments of *U. longissima* in each quadrat were marked with pins of different colors. The number and the length of each marked *U. longissima* strand were recorded before and after a feeding visit.

### 2.4 Effect of the Monkey on the Biomass of *Usnea longissima*

Third, we measured the biomass of *U. longissima* at the five sites (one current habitat and four historical habitats) in 73 sampling quadrats to test the long‐term interaction between *U. longissima* and the monkey. From October 2018 to March 2019, sampling quadrats were set up in H‐0, H‐10, H‐20, H‐30, and H‐40. In each of the five habitats, we set up quadrats (20 m × 20 m) in five elevation zones with an interval of 100 m from 3000 to 3400 m a.s.l. Due to topographical restrictions, quadrats in H‐40 were established at the following elevations: 3000 to 3100 m at Tianchi and 3200 to 3400 m at Xi Shan. We divided mountain slopes into four directions: north (0 ± 45 degrees), east (90 ± 45 degrees), south (180 ± 45 degrees), and west (270 ± 45 degrees). Based on our observation, the southern slopes are heavily influenced by human activities and largely populated by Yunnan pine (*Pinus yunnanensis*) forest, which is generally avoided by the monkeys. In every elevation zone, we placed one quadrat on a northern slope, one on an eastern slope, and one on a western slope. Fifteen quadrats were established in H‐0, H‐10, H‐20, and H‐30, respectively. In H‐40, only 13 quadrats were established because there is no western slope at 3100 m and no eastern slope at 3200 m.

We randomly selected five tall trees (three conifers and two broad‐leaved trees) from each of the 73 quadrats. The canopy of each tree was also divided into 24 segments, as described in the previous section. A small quadrat with a size of 5 × 5 cm was randomly placed in each segment. There were 8760 small quadrats (24 segments trees×73 quadrats). The length of each *U. longissima* filament from the root to the tip in each quadrat was recorded, and the biomass of *U. longissima* was calculated as No. of filaments×Average length. This metric provides a proxy for lichen biomass but does not account for variation in thallus thickness or density, which may affect total biomass estimates.

### 2.5 Effect of the Monkey on the Dispersal of *Usnea longissima*

Fourthly, we tested whether the monkeys' consumption of lichens promotes the dispersal of *U. longissima* by comparing the dispersal number and length of filaments between feeding ground and non‐habitat of *R. bieti* through a net trap experiment. Two 20 × 20 m quadrats were established in a conifer forest at Lasha Shan, one within H‐0 and another near H‐0. We randomly selected five tall trees in each of the two quadrats, and vertically divided the canopy of each tree into three layers (upper, middle, and lower) and horizontally into two layers (inner and outer). The canopy of each tree was thus also divided into 6 segments. A small quadrat with a size of 5 × 5 cm was randomly placed in each layer with a westerly direction. There were 60 small quadrats (6 segments trees×2 quadrats). We measured the length of each filament of *U. longissima* in each small quadrat. We placed five 2 m × 2 m net traps on the ground in the center and each corner of each quadrat. After 10 days, we collected *U. longissima* from each net trap and measured their length.

### 2.6 Effect of the Monkey on the Growth Rate of *Usnea longissima*

**FIGURE 4:**
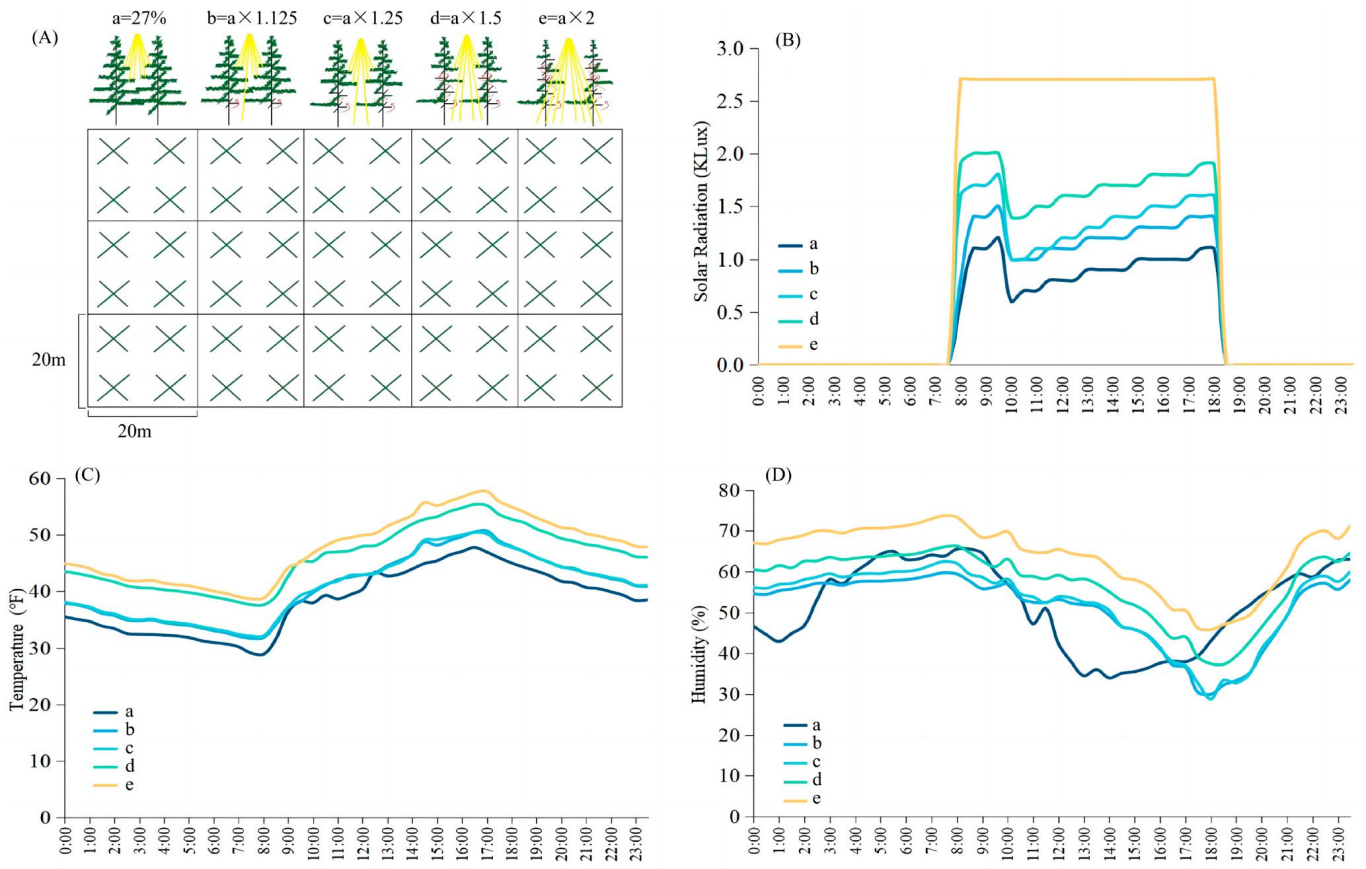
Simulated experiment to measure how the solar, heat, and water conditions vary with the size of canopy gaps. (A) Five groups of quadrats with 3 repetitions were set up in the same area: A‐original size of canopy gaps; b‐the canopy gap size was pruned to 1.125 times of a; c‐the canopy gap size was pruned to 1.25 times of a; d‐the canopy gap size was pruned to 1.5 times of a; e‐the canopy gap size was pruned to 2 times of a. Daily fluctuation of (B) Solar, (C) heat, and (D) water of under‐canopy showed similar trends and all increased with the size of canopy gaps.

Finally, we artificially pruned branches to simulate the expansion of the canopy gap by monkeys (the author's unpublished data), and cut the *U. longissima* short to simulate the monkey's foraging behavior, to test the effect on the growth rate of *U. longissima* . In November 2018, we set a 100 × 60 m sample area in Jiayan at a.s.l. 3300 m on the north slope. We set up fifteen quadrats (20 × 20 m) in the sampling area. In each quadrat we conducted 3 repetitions of each of five treatments: (a) original canopy gap without pruning, that is the control group (the size of the original canopy gap was 27%); (b) the size of canopy gap was pruned to 1.125 times the original size of canopy gaps; (c) the size of canopy gap was pruned to 1.25 times the original size; (d) the size of canopy gap was pruned to 1.5 times the original size; (e) the size of canopy gap was pruned to 2 times the original size (Figure 4A). We modulated the canopy size of each quadrat by pruning branches of each tree with DBH greater than 20 cm, starting from the bottom branches and moving upward in a counterclockwise direction. Referencing the method of Frazer et al. (1999), the fisheye lens was used to take 3 photos at a position of 1.5 m from the ground in the center of each quadrat, and then the photos of the hemispheric surface were analyzed with the Gap Light Analyzer software to determine the size of the canopy gap. In total, 90 photos of the hemispheric surface were taken. We also installed a photometer and hygrometer in the center of the quadrat. Solar radiation, heat (temperature), and water (humidity) conditions were recorded every 30 min throughout the day from February 2019 to March 2020.

Four trees were selected from each quadrat. The canopy of each tree was vertically divided into three vertical layers: up, middle, and low; and two horizontal layers: inner and outer with the trunk as the center. Therefore, each plant was divided into six layers. Two adjacent 5 × 5 cm small quadrats were set in each layer, in one quadrat filaments of *U. longissima* were uncut, and in the other one filaments were cut to 5 cm to simulate the feeding behavior of monkeys. The *U. longissima* in the quadrat was marked with colored I‐pins in the order of red, yellow, orange, blue, green, purple, and white for filament discrimination, and the length of the marked *U. longissima* was measured again one year later.

### 2.7 Statistical Analysis

**FIGURE 5:**
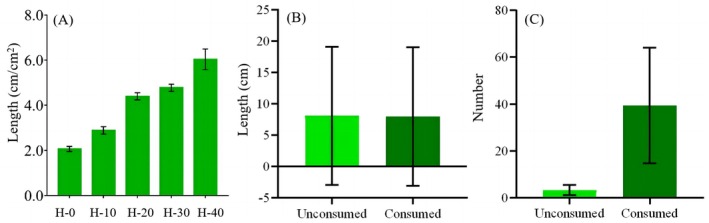
Feeding behavior of the black‐and‐white snub‐nosed monkey ( *Rhinopithecus bieti* ) reduces the biomass and increases the number of dispersal thallus of *Usnea longissima* . (A) The biomass of *U. longissima* in the current habitats was significantly lower than in historical habitats. (B) The length of dispersal thallus was not significantly different between the control area and the habitat. (C) Much more *U. longissima* were collected on the ground in the monkeys' habitat than in the control area. Error bars indicate ±SEM H‐0 is the currently used habitat by black‐and‐white snub‐nosed monkeys; H‐10, H‐20, H‐30, and H‐40 are the four historical habitats from which the monkeys vanished around 10, 20, 30, and 40 years ago, respectively.

**FIGURE 6:**
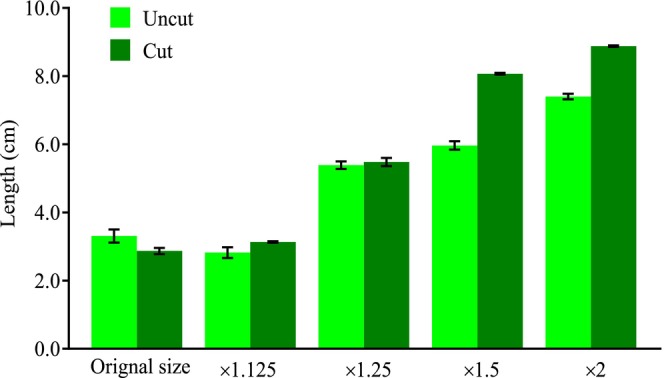
Growth rate of *U. longissima increases* with the size of canopy gaps, and the consumed thallus has a significantly higher growth rate than the unconsumed ones. Original size‐natural canopy gap, the control group; ×1.125‐the size of canopy gap was pruned to 1.125 times the original size of canopy gaps; ×1.25‐the size of canopy gap was pruned to 1.25 times the original size; ×1.5‐the size of canopy gap was pruned to 1.5 times the original size; ×2‐ the size of canopy gap was pruned to 2 times the original size. In each of the five groups, two treatments on *U. longissima* were contained: uncut and cut. We cut *U. longissima* short to simulate the ones consumed by the foraging monkey. Error bars indicate ±SEM.

Considering the sample sizes of the data and whether they conform to a normal distribution, we conducted statistical tests on various variables using the following methods: Analysis of variance (ANOVA) for more than two samples and for variables with a large sample size (*n* > 30) and with a normal distribution: the consumption amount of lichen by the monkey among various canopy layers, and the biomass of lichen among five habitats. Pearson correlation test for the lichen biomass and the time elapsed since the monkeys vanished (Figure 5A). The Mann–Whitney *U* test for variables with a small sample size (*n* < 30), discontinuous data, or non‐normal distribution: the length and number of dispersal lichen filaments between habitat and non‐habitat (Figure 5B,C). Welch two‐sample *t*‐test for variables with a large sample size (*n* > 30) and with a normal distribution: the consumption amount of lichen by the monkey between outer and inner canopy layers, and the growth rate between cut and uncut lichen filaments (Figure 6). All confidence levels were considered as 95%. All analyses were performed in the R platform (R Core Team 2022) version 4.1.0 with the package “stats” and RStudio environment (RStudio Team 2023).

## 3 Results

### 3.1 The Lichen *U. longissima* Increased the Rate and Extent of Tree Branch Dying

A total of 960 records of the health status of 160 tree branches were collected in the four quadrats in Jiayan. In the first monitoring period, all sampled branches were healthy. The total number of unhealthy branches increased over time in all four quadrats, and the number of unhealthy branches in the experimental groups receiving the four treatments was higher than in the control groups (76 vs. 32 in the last monitoring period).

The branches covered with *U. longissima* reached higher levels of desiccation than branches without *U. longissima* in all four treatments; from treatment A–D, the branches became faster and more desiccated (Figure 3A–D). For instance, the proportion of desiccated branches in the four treatments between lichen‐uncovered and covered branches is 0% versus 50%, 15% versus 80%, 15% versus 80%, and 100% versus 100%, respectively, in the 12th month. Especially, the branches died more quickly in the treatment group where *U. longissima* on the main branch was girdled at its root than in the other experimental groups. All the branches died in the 12th month in this treatment group, but not in the other treatment groups. Even the healthy main branches without side branches or bark removal also became desiccated after the lichens were hung on.

### 3.2 Partial Consumption of Lichen by the Monkey

A total of 2981 *U. longissima* filaments were measured in Lasha Shan, of which 932 were fed on by the monkeys, accounting for 31% of the total number. Of the 932 *U. longissima* filaments that the monkeys fed on, 289 were consumed entirely and 643 partially. This shows that the monkey did not deplete *U. longissima* during a visit to a lichen‐bearing tree (Table 2). The average length of all filaments of *U. longissima* combined per tree before being fed on by the monkey was 16.30 ± 3.72 m, while the average length after being fed on was 13.95 ± 3.26 m. Therefore, the average amount of lichen consumed by the monkeys in a single tree was equivalent to a segment extending to 2.35 ± 0.57 m in length. Thus, the monkey group consumed an average of 14.42% of the biomass of *U. longissima* during a single visit to a feeding tree, including that which were eaten completely and partially.

**TABLE 2:** The effect of feeding behavior of *Rhinopithecus bieti* on *Usnea longissima* in various canopy layers within a tree.

Location of *U. longissima* within a tree	Average no. of *U. longissima* filaments before a feeding visit	Average no. of completely eaten *U. longissima* filaments (%)	Average no. of partially eaten *U. longissima* filaments (%)
Upper canopy	22.12 ± 4.50	1.76 ± 0.12 (8.00)	5.78 ± 1.18 (26.13)
Middle canopy	20.82 ± 4.32	2.40 ± 0.22 (11.53)	4.00 ± 0.85 (19.21)
Lower canopy	16.68 ± 2.58	1.62 ± 0.24 (9.71)	3.10 ± 0.74 (18.59)
Outer layer	30.48 ± 7.46	2.30 ± 0.69 (7.55)	7.94 ± 1.41 (26.05)
Inner layer	29.14 ± 7.36	3.48 ± 0.87 (11.94)	4.94 ± 0.73 (16.95)

Our analyses showed that the monkeys consumed the largest amount (1.25 ± 0.23 m per tree) of *U. longissima* in the upper canopy and the smallest amount (0.37 ± 0.06 m per tree) in the lower canopy (ANOVA, *F* = 86.27, *p* < 0.0001). The total amount of lichens consumed (including both completed and partially removed filaments) in the outer layer of the canopy was not significantly higher than in the inner layer (1.14 ± 0.19 m vs. 1.04 ± 0.15 m per tree; *t* = −1.04, *p* = 0.200). While partially eaten *U. longissima* were recorded at a greater amount in the outer layer of the canopy than in the inner layer (0.64 ± 0.18 m vs. 0.34 ± 0.09 m per tree; *t* = −4.082, *p* < 0.0001).

### 3.3 Reduced Lichen Biomass in Current Monkey Habitats Compared to Historical Habitats

We measured 34,798 *U. longissima* filaments in the five sites (Table 3). The biomass of *U. longissima* in the current habitat was significantly lower than in the four historical habitats (ANOVA, *F* = 43.25, *p* < 0.0001), and the biomass of *U. longissima* in H‐0 was less than half of that in H‐20 (*t* = 30.25, *p* < 0.0001), H‐30 (*t* = 41.93, *p* < 0.0001), and was nearly one third of H‐40 (*t* = 7.67, *p* < 0.0001). The more time elapsed since the monkeys vanished in a particular habitat, the greater the biomass accumulation of *U. longissima* (*R* ^2^ = 0.78, *p* < 0.0001; Figure 5A).

**TABLE 3:** Measurements of biomass of *Usnea longissima* in current and historical habitats of *Rhinopithecus bieti* .

Items	Subitems	H‐0	H‐10	H‐20	H‐30	H‐40
No. of filaments		3363	4169	5614	5749	6751
Average length of *Usnea longissima* (cm)	Total	23.92 ± 4.96	28.49 ± 5.32	30.51 ± 5.57	32.38 ± 5.75	34.88 ± 5.88
	Upper layer	27.49 ± 4.86	33.85 ± 5.36	36.49 ± 4.72	38.08 ± 4.67	40.36 ± 5.67
	Middle layer	23.20 ± 4.88	27.92 ± 5.11	29.30 ± 4.69	30.64 ± 5.26	32.94 ± 5.62
	Lower layer	19.89 ± 4.65	22.69 ± 4.74	24.72 ± 4.86	28.06 ± 5.06	30.73 ± 5.69
	Inner layer	22.06 ± 5.30	27.01 ± 5.75	30.30 ± 6.78	32.56 ± 6.11	34.38 ± 6.54
	Outer layer	25.67 ± 5.27	29.86 ± 5.71	30.72 ± 6.59	32.21 ± 6.97	35.35 ± 6.21

### 3.4 Enhanced Dispersal Efficiency of Lichen in Monkey‐Inhabited Versus Noninhabited Sites

The average length of a single filament of *U. longissima* within the monkeys' habitat in Lasha Shan was 24.59 cm (*n* = 53), whereas in non‐habitat, it was 39.39 cm (*n* = 103). Outside the habitat, we retrieved 16 dispersed *U. longissima* from the net traps with a total length of 129.31 cm, whereas there were 197 dispersed filaments inside the habitat, with a total length of 1570.00 cm. The length of dispersal filaments in the feeding ground were not significant different than those where the monkeys were not feeding (Manner–Whitney test, *U* = 1292, *p* = 0.2337; Figure 5B), whereas the dispersal number of *U. longissima* filaments in the feeding ground was significant higher (Mann–Whitney test, *U* = 0.5000, *p* = 0.0080; Figure 5C).

### 3.5 Stimulated Growth Rates in Cut Lichen Specimens Relative to Uncut Controls

We found that the wider the canopy gap, the higher the solar radiation, the higher the temperature, and the higher the humidity (Figure 4B–D), though the fluctuation trends of each variable across a day in quadrats of various canopy gap sizes were similar. These results demonstrate the significant effects that canopy gaps exert on solar, heat, and water conditions in areas below the canopy.

We recorded 2102 length data of *U. longissima* , including 977 artificially cut and 1125 uncut filaments. The results showed that the growth rate of *U. longissima* increased linearly with the size of canopy gap, and cut ones grow faster than uncut ones (Figure 6). The average growth length of *U. longissima* that had been cut was 3.82 ± 0.65 cm, while that of the uncut *U. longissima* was 2.99 ± 0.51 cm, showing a significant difference (*t* = −14.846, *p* < 0.001).

## 4 Discussion

Based on our study, it has been observed that the snub‐nosed monkey's main food, *U. longissima* , exhibits harmful effects on trees; this may indicate the lichen was a parasite rather than an epiphyte, although microscopic evidence of haustorium‐like structures is needed to confirm this hypothesis. Whereas in general, lichens have been default assumed to be epiphytes (Esseen et al. 2022). Lichens make a distinct contribution to ecological processes and ecosystem function, which has greatly proven in recent decades (Porada et al. 2014, 2018; Payette and Delwaide 2018; Porada and Giordani 2022; Esseen et al. 2022). A wide range of consumer or user organisms depend on lichens (see Asplund and Wardle 2017). In addition, many lichens are sensitive to environmental disturbance, so they are consequently used as indicators of ecosystem integrity and global change (Allen et al. 2019; Ellis 2019; Ellis and Coppins 2019; Giordani et al. 2020; Esseen et al. 2022). However, there is hardly any research to prove whether lichens are epiphytes or parasites and their effect on host plants. In fact, few studies have noticed that lichens may harm trees. For example, hair‐like lichens (*Alectoria, Bryoria, Usnea*) were very abundant a century ago; they were assumed to reduce tree growth or even kill trees (Romell 1922). In light of these statements, there is a great need for accurate investigations to clarify whether lichens are epiphytes or parasites. Microscopic structures of *U. longissima* and the colonization process should be studied further. Heightened attention should be directed toward the potential repercussions, particularly in mature forests where lichen populations are stable and biomass accumulates significantly.

The rare primate *R. bieti* and its main diet *U. longissima* may have formed a mutualistic symbiotic relationship. The monkeys appear to help regulate *U. longissima* biomass, potentially mitigating excessive accumulation that could harm forest health. The lichen *U. longissima* is dispersal‐limited, as it primarily propagates through thallus fragments with limited wind dispersal capacity (Esseen et al. 2023). So the lichen is quite old‐growth associated and requires extensive and continuous tracts of suitable habitat (Esseen et al. 2023). Given its limited dispersal ability and high habitat specificity, large‐scale treefall events could lead to local extirpation of *U. longissima* (Strother et al. 2022; Esseen et al. 2022; Esseen and Ekström 2023). The monkeys consumed the lichen to reduce its harm to trees and sustain old forest, in return may also avoid the lichen's dispersal limitation and refugia lost.

Regionally, the monkey *R. bieti* potentially contributes to the dispersal of *U. longissima* by promoting the growth of saplings (unpublished data) and increasing the dispersed thallus. This ecological relationship parallels the interaction between the golden snub‐nosed monkeys *R. roxellana* and lichens *U. diffracta* (Qin et al. 2025), suggesting a potentially conserved primate‐lichen dynamic in montane forest ecosystems. Lichens have a high growth potential due to their numerous small fragments and symbiotic reproductive bodies (McCune et al. 1996; Gauslaa et al. 2007; Rolstad and Rolstad 2008; Jansson et al. 2009). These reproductive bodies can quickly colonize young trees (Hyvärinen et al. 1999; Clerc 2011). At the same time, *U. longissima* exhibits better growth performance in well‐lit forest gaps (Gauslaa et al. 2007; Storaunet et al. 2008, 2014; Esseen et al. 2023), as also confirmed in this study. Besides, the damage caused by *U. longissima* to tree branches and the foraging behavior of monkeys collectively widen the canopy gaps and increase light availability, improving growth. The lichen becomes shorter while exhibiting an accelerated growth rate after monkeys feed on it (based on our artificial cutting experiment, which may not fully replicate the biological effects of actual monkey consumption). Similarly, the specialized diet of giant pandas is proven to help the clonal regeneration of bamboo populations by improving the density and the recruitment rate of shoots (Zhang et al. 2019). Thus, their potential mutualistic symbiotic relationship may help maintain the populations of both the lichen and the monkey and contribute to the sustainability of the forest ecosystem. However, long‐term population monitoring and experimental manipulations are needed to confirm this hypothesis.

The specialized diet of certain species also underscores their ecological fragility. For giant pandas, overgrazing, insect, and warmer climate would threaten the sustainable utilization of their staple bamboo resources, thereby compromising its population (Zhang et al. 2019). Hummingbirds serve as a classic example of dietary specialization, and scientists are uncertain about how plant shift will mediate hummingbird survival under changing environmental conditions (Barreto et al. 2024). Similarly, the fact that this lichen accounts for up to 80% of the diet composition also implies potential risks. If the population of this lichen decreases due to climate change, forest degradation, or pathogen invasion, the monkey may face the threat of food shortage and even affect its population survival. Similar dietary relationships arise in other primate ecosystems. The golden snub‐nosed monkey, for example, relies heavily on lichen (comprising over 40% of its winter diet) due to its protein‐rich composition (Zhao et al. 2020), while their ecological relationships also need to be learned further. This dependence may exacerbate the vulnerability of endangered species when facing environmental changes.

Future research should focus on the correlation between the population dynamics of *U. longissima* and the survival of the monkey *R. bieti* , assess the alternative food sources within its geographical distribution range, and adopt management methods that increase food‐producing plants. Efforts should also be made to quantify the relationship between the proportion of lichens in the diet of the monkey and the abundance of other food plants in multiple areas. And the multi components interaction patterns under environment change should be studied to further clarify the ecosystem functions of the monkey.

Our research has several limitations. Although field observations suggest that dispersal fragments increase where monkeys feed, we did not experimentally track the long‐term survival and establishment success of these dispersed thalli. Additionally, our comparison between historical and current lichen biomass relies on recent survey data; while we observe a decline in lichen presence coinciding with reduced monkey populations, other unaccounted historical factors may also contribute to the observed patterns. We further acknowledge that the hypothesis of lichen parasitism on trees lacks histological or microscopic validation. Finally, our experimental simulation of monkey foraging behavior involved manually cutting lichen, which necessarily simplifies the complexity of natural feeding and may not fully replicate the mechanical or behavioral nuances of actual primate foraging. In future studies, we aim to conduct more in‐depth and comprehensive investigations to clarify the underlying mechanisms.

Forest management should carefully consider the intricate interactions among lichens, trees, and consumers. Enhancing research on the ecological impacts of other prevalent tree‐dwelling lichen species and their interaction with multiple taxa are imperative. This understanding, along with the development of conservation strategies and sustainable harvesting practices, especially for lichens used in traditional medicine (Chang et al. 2016; Crawford 2019; Wang et al. 2021) is essential.

Furthermore, this study found that the interactions among ecosystem components are diverse, multiprocess, and of complex nature, demanding focused attention in ecosystem management strategies. Such complexity is particularly critical when addressing key components that may shape the overall ecological stability. However, existing research often simplifies these interactions into isolated processes, dichotomizing them as purely beneficial or detrimental, which could significantly impact the understanding of sustainable ecosystem management and maintenance. For example, the relationship between reindeer and lichen in tundra ecosystems is often focused on the process of reindeer grazing on lichen (Scotter 1967; Danell et al. 1994; Heggberget et al. 2002; Sundset et al. 2008, 2010; Dominy et al. 2023). While reindeer grazing also modifies soil microclimate (Stark et al. 2010), prolonged dietary specialization has driven evolutionary adaptations in reindeer vision and digestive systems (Dominy et al. 2023). These dynamics parallel those observed in the present study—interactions between monkeys and lichens—where symbiotic relationships may also involve unseen evolutionary trade‐offs.

This pattern mirrors human relationships with crops like wheat and rice, where co‐evolutionary dynamics have reshaped landscapes, biodiversity distributions, and human societies. These examples underscore that ecosystem management must transcend oversimplified categorizations of “beneficial” or “harmful” interactions. Instead, future strategies should prioritize comprehensive investigations into the interconnected roles of keystone components. By adopting a holistic framework, researchers and managers can better anticipate ecosystem responses to perturbations and avoid unintended consequences of ecological mismanagement.

## Author Contributions

**Na Li:** data curation (lead), formal analysis (lead), methodology (lead), software (lead), writing – original draft (lead), writing – review and editing (lead). **Hao‐Han Wang:** data curation (lead), formal analysis (lead), funding acquisition (equal), investigation (lead), methodology (lead), software (lead), validation (equal), writing – original draft (equal). **Yan‐Peng Li:** investigation (equal), project administration (equal), visualization (equal). **Cyril C. Grueter:** methodology (equal), writing – original draft (equal). **Hui‐Ming Xu:** investigation (equal). **Zhi‐Pang Huang:** formal analysis (equal), funding acquisition (equal), investigation (lead), project administration (lead), resources (equal), validation (equal), visualization (equal). **Wen Xiao:** conceptualization (lead), methodology (lead), project administration (equal), resources (lead), supervision (lead), validation (lead).

## Funding

This study was supported by the National Natural Science Foundation of China (32371567; 32360137; 31860164); Talent and Platform of Science and Technology in Yunnan Province Science and Technology Department (202105AM070008; YNWR‐QNBJ‐2019‐262; YNWR‐CYJS‐2018‐052).

## Conflicts of Interest

The authors declare no conflicts of interest.

## Data Availability

All data associated with this study are publicly available. The raw data supporting the findings of this research are openly accessible in Dryad at https://doi.org/10.5061/dryad.0p2ngf2fn.
